# Molecular Residual Disease in Non-Small Cell Lung Cancer: Technology or Patient Outcomes?

**DOI:** 10.3390/jpm16090454

**Published:** 2026-08-29

**Authors:** Paul R. Walker

**Affiliations:** 1 Emeritus Faculty, Division of Hematology/Oncology, Brody School of Medicine at East Carolina University, Greenville, NC 27834, USA; walkerp@ecu.edu; 2 Circulogene, Pensacola, FL 32502, USA

**Keywords:** molecular residual disease (MRD), circulating tumor DNA (ctDNA), tumor-informed assay, plasma-informed assay, variant allele fraction (VAF), level of detection (LOD), non-small cell lung cancer (NSCLC)

## Abstract

Molecular residual disease (MRD) testing approaches with plasma next-generation sequencing (NGS) testing to identify circulating tumor DNA (ctDNA) in resectable-stage non-small cell lung cancer (NSCLC) are evolving. The MRD concept is to better guide perioperative systemic treatment and identify recurrent NSCLC before symptomatic radiographic recurrences. Multiple tumor-informed assays are available with technology driving lower levels of ctDNA detection. However, it remains unclear that individual patients derive survival outcome benefit from MRD testing. NSCLC tumor biology of spatial heterogeneity, early parallel metastases, and recurrence clonal evolution can impact tumor-informed approaches irrespective of specific assay level of ctDNA detection. Clinical decision making guided by tumor-informed MRD testing to date have been limited by recurrence risks of up to 20% when landmark MRD-negative, improved outcomes benefit of adjuvant treatment even when landmark MRD-negative, and lead times with longitudinal MRD-positive conversion of several months or longer before overt radiographic recurrences with no proven strategy of survival benefit with intervening treatment. Cautionary tumor biology and clinical issues remain in the clinical utility of tumor-informed MRD testing in resected NSCLC. These need to be clarified with certainty before MRD testing should step beyond a technology-driven prognostic recurrence risk indicator before becoming an absolute clinical guide to meaningfully impact individual patient management and outcomes.

## 1. Introduction

Molecular residual disease (MRD) circulating tumor DNA (ctDNA) testing is evolving in the perioperative management of resectable-stage non-small cell lung cancer (NSCLC) [1,2,3]. By detecting the presence or absence of plasma ctDNA mutations, gene rearrangements, or copy number variations after perioperative systemic therapy and/or curative-intent surgery, MRD assessment can identify patients at high risk or low risk of recurrence. Plasma ctDNA MRD can be assessed by two different testing approaches: a personalized mutation panel developed for each individual based upon the primary tumor mutations identified in tissue (tumor-informed assay) or a larger multi-gene panel not limited to specific primary tumor tissue mutations (tumor-agnostic; tumor-naïve; or plasma-informed assays, which will be used in this review).

The technology to support perioperative MRD ctDNA testing continues to improve. As a general comparison, there are basic differences beyond panel size in the types of MRD assays. Tumor-informed assays are multiplex polymerase-chain-reaction-based testing allowing deeper ctDNA variant allele fraction (VAF) level of detection (LOD) capability than plasma-informed next-generation sequencing (NGS) ctDNA assays. Conversely, tumor-informed MRD assay testing is limited to the primary tumor clones. Plasma-informed MRD assays are not limited to primary tumor clones and can also identify additional ctDNA mutations from any parallel evolution of early metastatic clones, clonal and sub-clonal evolution of recurrence and treatment-resistant pathways of any perioperative systemic treatment. There is also a notable turnaround time difference and thus different clinical utility in the two types of assays. The time for development of a tumor-informed assay takes weeks to develop after the tissue has undergone a surgical pathologic evaluation limiting it to a post-surgical assessment and one-time decision making of adjuvant treatment. It is not available to guide any neoadjuvant treatment decision making. Plasma-informed assays have a much faster initial turnaround time of days and can guide neoadjuvant treatment decision making as well as assess ctDNA response to the neoadjuvant treatment and guide adjuvant treatment decisions.

MRD testing is benchmarked into two testing phases, each with distinctly different clinical utility and decision-making purposes. Landmark MRD testing is defined as the initial testing time point after surgery impacting adjuvant treatment decision making. Longitudinal MRD testing is ongoing surveillance testing over a defined period of follow-up to detect early recurrences before overt radiographic recurrent cancer.

Technology drives MRD assays. But have MRD assays driven improved patient outcomes? MRD-positive is cancer-positive, but MRD-negative is not always cancer-negative. Concepts of treatment escalation or de-escalation and early cancer recurrence detection based upon MRD detection are taking hold in the clinic. Yet questions persist unanswered on whether MRD testing improves individual patient outcomes. Without awareness of the potential impact of tumor biology and limitations of MRD assays, the assumption of MRD assays being clinically absolute could lead to individuals with resected NSCLC not receiving an optimal personalized multi-disciplinary treatment approach and outcome. This narrative review will focus not on specific MRD assays or MRD technology advances but on the perspective of limiting tumor biology questions and clinical issues impacting tumor-informed MRD assay use in the clinic for individual patients with NSCLC.

## 2. Does Tumor Heterogeneity Impact Tumor-Informed MRD Assays?

Spatial inter-tumoral and intra-tumoral heterogeneity is a recognized limitation of tissue molecular testing in advanced NSCLC [4]. Cancer accumulates somatic mutations as it evolves. Molecular heterogeneity between primary and metastatic sites is tumor biology evident in the clinic with discordant tumor site responses. Discordance of epidermal growth factor receptor (*EGFR*) mutations of 10–20% have been noted between primary tumors and metastatic sites [5]. Up to 40% of paired primary tumor and metastatic sites demonstrated a discordant molecular profile with tissue NGS testing [6].

Tumor heterogeneity can be particularly impacted based upon the number of areas sampled. Multiregional sequencing of primary and associated metastatic sites of clear cell renal carcinoma biopsies revealed 63–69% of somatic mutations were not identified at a single biopsy site and 25–50% of somatic variants in 19 single biopsies were private mutations not detected in other biopsy sites within the same tumor [7].

Studies in advanced NSCLC assessing molecular testing in parallel tissue samples and plasma-informed NGS assays have consistently highlighted the limitations of tissue molecular intratumoral heterogeneity. In advanced NSCLC studies, tissue testing identified only 55–67% of druggable biomarkers, whereas plasma NGS outperformed tissue identifying 80–87% of druggable alterations in these same patients [8,9,10,11]. In a large patient cohort of 1127 advanced NSCLC patients, 25% of genomic alterations identified in the plasma NGS were not found in time-matched tissue testing [12]. A rapid autopsy series in advanced gastrointestinal tumors demonstrated significant inter-tumoral and intra-tumoral heterogeneity across metastatic sites best identified with plasma NGS compared to tumor site biopsy sampling [13].

Similar findings have been noted in other solid tumors. In a breast cancer study of 252 patients undergoing matched tissue and plasma-based molecular testing with a plasma-informed assay with a reported analytic sensitivity of 0.1%, plasma ctDNA identified 78% actionable mutations compared to tissue with 50% actionable mutations [14]. In concurrent molecular testing in breast and prostate cancer, 20% of guideline-based actionable findings were only identified by plasma NGS testing [15]. In the ROME study of 1794 patients with advanced solid tumors, concordance was present in 49% of patients with alterations exclusively detected in tissue (35%) or liquid (16%), further supporting the need for plasma-informed assay NGS testing to identify genomic alterations not identified in tissue [16].

Circulating tumor cells have also demonstrated a dynamic evolution of single-cell genomic, transcriptomic and proteomic discordance and diversity between primary and metastatic sites [17,18,19].

Spatial intra-tumoral heterogeneity within primary tumors has also been noted. In 20 patients with resected anaplastic lymphoma kinase (*ALK*)-fusion-positive NSCLC, 45% (9 out of 20) of patients had areas of wild-type *ALK* within the same tumor specimen and 32% (11 out of 34) of pathologically sampled areas were *ALK*-fusion-negative [20]. Multiregional whole-exome sequencing and/or whole-genome sequencing testing in 25 spatially distinct regions in seven resected lung adenocarcinoma primaries demonstrated heterogeneity in all seven patients with a median of 30% heterogenous mutations [21].

The evidence supporting inter-tumoral and intra-tumoral heterogeneity comes from the metastatic NSCLC setting with expected genomic instability and clonal evolution impacting ctDNA shedding and VAF. This degree of underlying advanced tumor biology findings may not have evolved in the occult post-surgical MRD state. If not present, this would limit the impact of tumor heterogeneity on tumor-informed MRD assays. However, the demonstrated primary tumor spatial intra-tumoral heterogeneity may narrow the development of the MRD assay to just the sampled tumor area. Developed inter-tumoral heterogeneity between the primary tumor and metastatic sites upon recurrence could limit the completeness of MRD monitoring.

## 3. Can Tumor-Informed MRD Assays Miss Metastatic Clones in NSCLC?

There are two recognized models of metastases: a linear progression model and a parallel progression model [22]. In the linear model, metastases occur late in tumorigenesis with minimal divergence between primary and metastatic clones. In the parallel model the clone or subclone disseminates early from the primary tumor with a potentially high degree of divergence between the primary and metastatic clones [23].

The seminal Tracking Non-Small-Cell Lung Cancer Evolution through Therapy (TRACERx) patient cohort provides insights into ctDNA in resected NSCLC. Two primary tumor pathology specimen regions separated by 0.3 cm to 1 cm were undertaken with a tumor-informed customized ctDNA assay tracking a median of 200 genomic alterations [24]. In the expanded TRACERx 421 patient cohort, 126 out of 421 patients developed metastatic disease. Clonal mutations in all primary tumor regions were absent in the metastatic site in 25.4% (32 out of 126) and metastasis unique driver mutations were identified in 33.3% (42 out of 126) [25]. TRACERx researchers viewed these findings suggesting early metastatic divergence prior to initial diagnosis occurring when the tumor diameter was less than 8 mm supporting the parallel progression model of metastases in these patients with early-stage NSCLC [25].

Clonal evolution will occur with systemic cancer treatment. Whole-exome sequencing in 457 primary tumors and metastatic samples in 136 patients with breast, colorectal, and lung cancer noted mutational differences in treated and untreated metastasis. Treated metastasis often harbored private mutations, whereas untreated metastasis did not [26]. TRACERx data also noted prior platinum chemotherapy contributed to tumor heterogeneity and clonal evolution [25].

Clonal evolution with cancer progression is well established in metastatic NSCLC. Resistant pathways evolve with active treatment. Repeat plasma NGS testing in patients progressing on immune-based treatment in the metastatic setting identified new druggable targets not identified in pre-treatment plasma NGS testing in 26% of patients [27]. Acquired treatment resistant pathways in NSCLC will vary based upon the type of initial treatment. A cohort of advanced NSCLC patients treated with immune-based therapies demonstrated 27.8% new mutations at the time of resistance and with targeted TKI-treated patients acquiring 32.6% new mutations [28].

In the NSCLC post-surgical recurrence setting, plasma-informed MRD assays identified more actionable genomic alterations than tumor-informed assays. In a systemic meta-analysis of 30 studies involving 3287 patients with post-operative NSCLC MRD testing, landmark tumor-agnostic MRD assays identified 17.43% Level 1 and 42.66% Level 2 mutational clinical significance based upon the categorization of the Association for Molecular Pathology, American Society of Clinical Oncology and College of American Pathologists. This compared to 0.34% Level 1 and 0.84% Level 2 findings with tumor-informed assays [29,30].

MRD detection of tumor-informed assays may be limited by discordant parallel metastatic clones and the clonal evolution of progressed metastases missing precision oncology treatment of a cancer recurrence.

## 4. Can False-Positive or False-Negative Findings Impact Plasma-Informed MRD Assays?

Plasma-informed assays are less sensitive with a higher LOD than tumor-informed assays, resulting in more false-negative MRD results than the more sensitive and lower LOD of tumor-informed assays. In addition, plasma-informed assays can be subject to confounding plasma cell-free DNA alterations that are not tumor-related. These findings can generate false-positive results. Age or prior treatment-related clonal hematopoiesis of indeterminate potential (CHIP) mutations, germline mutations and other tissue cell-free DNA origins can result in false-positive MRD detection [31]. Bioinformatic exclusion of known classic CHIP mutations and VAF in the 50% range to exclude germline mutations or analytic myeloid testing to exclude CHIP and germline mutations can overcome these false positives, providing tumor origin certainty.

## 5. Do Landmark MRD-Negative Patients Recur?

The TRACERx patient cohort outcomes demonstrated that 20% of landmark MRD tumor-informed negative patients became MRD-positive with longitudinal testing at a median of 357 days post-surgery (range 120–929 days) [32].

In the ADAURA trial, a post hoc comparison of 3 years of adjuvant osimertinib versus placebo in resected *EGFR* mutated NSCLC, a tumor-informed MRD assay was available in 220 of the 682 total patients. Similar to the TRACERx data, 23% (50 out of 220) of patients became MRD-positive after a negative landmark MRD result [33].

Gale et al. reported on 88 patients with early-stage NSCLC [34]. A pre-treatment tumor-informed MRD assay detected ctDNA in 24% stage I, 77% stage II, and 87% stage III, with 63% of the samples VAF < 0.1%. Longitudinal MRD testing detected ctDNA in 18/28 (64.3%) of patients who subsequently had a clinical recurrence. Landmark MRD-negative turned positive in 4/37 (10.8%) patients. Landmark MRD-negative had a negative predictive value (NPV) of 75.5%. Positive post-operative samples had a median VAF of 0.042%. Plasma ctDNA detection preceded clinical detection by a median of 7 months.

The LUNGCA-1 study monitored a tumor-informed 769 gene plasma NGS assay in 330 stage I-III resected patients before surgery and assessed landmark time points at 3 days plus 1 month after surgery [35]. Given most patients were stage I, 261 (79%) were ctDNA-negative pre-surgery with 15% of those patients recurring and 21% were ctDNA-positive, with 46% of those patients recurring. Post-surgery 303 (92%) patients were landmark MRD-negative, with 16% of those patients recurring.

Zhang et al. reported 43-month follow-up MRD outcomes in 261 patients with stage I (>2 cm)-III NSCLC undergoing curative surgery with landmark plasma-informed MRD testing 1 month after surgery (before starting any adjuvant treatment) and longitudinal testing every 3–6 months [36]. Both landmark and longitudinal MRD-detectable had equally strong positive predictive values (PPV) of 91.3% and 92.8%, respectively. However, landmark testing had an NPV of 76.5% compared to longitudinal testing of 93.2%.

Tumor-informed MRD studies in other solid tumors demonstrate similar patterns of recurrence in the landmark MRD-negative setting. In IMvigor011 in muscle-invasive bladder cancer, landmark tissue-informed MRD testing that was initially ctDNA negative subsequently tested MRD-positive in the longitudinal testing period in 41% of patients [37]. Landmark MRD-negative testing in stage III colon cancer was associated with 12–18% recurrences with tumor-informed MRD assays and 24–25% with a plasma-informed epigenomic methylation assay [38,39].

Certain metastatic compartment recurrences can be associated with MRD-negative results. In the lung adenocarcinoma TRACERx 421 patient cohort, intrathoracic recurrences and sanctuary central nervous system metastases were frequently MRD ctDNA-negative [32]. Another resected NSCLC patient cohort identified a 33.3% recurrence rate in landmark tumor-informed MRD-negative with 10/13 landmark MRD-negative patients with a locoregional or central nervous system compartment recurrence [40].

Remaining MRD-negative over time is associated with a minimal risk of recurrence in a resected NSCLC, just as negative imaging is associated with the same minimal risk of recurrence over that same time. Studies in NSCLC and other cancers consistently demonstrate that patients with landmark and longitudinal MRD-positive tumor-informed assay results have a universal risk of recurrence. However, even when landmark MRD-negative, resected NSCLC patients are still at a risk of recurrence over time. Landmark MRD-negative patient recurrence risks are highlighted in Table 1.

Longitudinal MRD negative patient outcomes do not imply or assume that landmark MRD-negative results will result in equivalent patient outcomes. Whether this is due to an MRD assay LOD issue, a balance of tumor biology ctDNA shedding with circulating nuclease clearance or compartmental ctDNA shedding as up to one-third of metastatic cancers have no detectable plasma ctDNA shedding, or the complexity of the metastatic niche and cascade beyond ctDNA is unclear [41,42,43].

**Table 1:** Highlighted studies of recurrence risks in resected NSCLC based upon landmark MRD testing. (LOD = level of detection; PPM = points per million; NR = not reported; NPV = negative predictive value; PPV = positive predictive value.)

Study	Patients	Stages	Landmark Testing	Assay Type	LOD/PPM ctDNA	NPV	PPV	MRD Negative Recurrence
TRACERx [32]	197	I-III	Within 120 days	Tumor-informed	0.008%/80	68%	93%	20%
ADAURA [33]	220	IB-IIIA	Within 10 weeks no chemo or after chemo	Tumor-informed	0.001%/10	78%	91%	23%
Gale [34]	88	I-III	2 weeks– 4 months	Tumor-informed	0.001%/10	75.5%	NR	10.8%
LUNGCA-1 [35]	330	I-III	3 days and 1 month	Tumor-informed	Research assay	NR	NR	16%
Zhang [36]	261	I-III	1 month or after chemo	Plasma-informed	Research assay	76.5%	91.3%	NR
Cui [44]	86	I-III	1 month	Tumor-informed Plasma-informed	Research assay	81.7% 75.0%	100% 75%	NR
Meta-analysis [29]	3287	I-III	Variable; 3 days after surgery up to within 3–4 months	Tumor-informed Plasma-informed	Variable assays	83% 77%	79% 65%	NR

## 6. Do Landmark MRD-Negative Patients Need Adjuvant Treatment?

That is one of the two pressing clinical utility questions of MRD testing. Can adjuvant therapy decision making be guided by landmark MRD testing? This question has complexities in the immune era treatment of resectable NSCLC with neoadjuvant/adjuvant approaches versus adjuvant-only approaches. Studies comparing neoadjuvant chemo-immune therapy with chemotherapy are consistently associated with higher pathologic complete responses, disease-free survival, and emerging overall survival outcomes over time [45,46,47,48]. Cross-trial comparison of studies in the adjuvant setting with chemo-immune therapy compared to chemotherapy have not consistently demonstrated a disease-free survival benefit [49,50,51]. Real-world retrospective data in resected stage II-III NSCLC from the National Cancer Database supports superior overall survival outcomes with neoadjuvant immune-based approaches compared to only adjuvant treatment [52]. This narrows a strict adjuvant treatment question to those not receiving neoadjuvant immune-based therapy. However, the neoadjuvant studies can still shed light on perioperative systemic treatment and landmark MRD status.

NSCLC studies with adjuvant chemotherapy demonstrate an outcome benefit in landmark MRD ctDNA-positive patients but not in MRD ctDNA-negative patients [35,36]. However, the survival outcomes in MRD-negative patients exceed the outcomes in MRD-positive patients receiving adjuvant chemotherapy. In the current era of chemo-immune or targeted perioperative therapy, MRD ctDNA-negative patients can still benefit from these perioperative systemic therapies.

In the phase III IMpower010 trial of resected stage II-IIIA NSCLC PD-L1-positive patients, similar hazard ratios (HR) of outcome benefit with the use of adjuvant sequential chemotherapy followed by atezolizumab was seen irrespective of landmark MRD status by a tumor-informed assay [53]. MRD-positive patients benefited from the addition of atezolizumab, with a median disease-free survival of 21.8 months with atezolizumab versus 7.2 months with chemotherapy alone (HR 0.58). MRD-negative patients also benefited from the sequential atezolizumab with markedly better outcome than MRD-positive patients. The median disease-free survival in landmark MRD-negative patients was yet to be met with atezolizumab compared to 52.6 months with chemotherapy alone (HR 0.60). There was the same relative benefit but with a far greater absolute benefit when landmark MRD-negative patients received adjuvant immune-based treatment.

In the ADAURA trial, most patients were landmark MRD-negative, 96% and 88% in the osimertinib and placebo arms, respectively. Yet the osimertinib-treated patients maintained an on-treatment MRD-negative state in 91% compared to 58% in the placebo arm supporting the use of adjuvant osimertinib irrespective of tumor-informed landmark MRD results. Most recurrences occurred upon osimertinib discontinuation, suggesting an ongoing treatment benefit even when landmark MRD-negative [33]. Two years after completing osimertinib MRD-negative patients had a 33% recurrence rate.

Neoadjuvant chemo-immune therapy trials have assessed ctDNA after neoadjuvant treatment before surgery but less so in the post-surgery setting. This limits a true post-surgical landmark MRD assessment. All but CheckMate 816 also had an adjuvant immune-checkpoint inhibitor treatment course after the neoadjuvant chemo-immune therapy, which would likely have an ongoing immune treatment effect on any residual micrometastatic cancer.

Clearance of ctDNA after neoadjuvant chemo-immune treatment is consistently associated with higher complete pathologic responses and survival outcomes but are still associated with recurrences. In CheckMate 816, clearance of ctDNA assessed by a tumor-informed assay was clearly better than a lack of clearance but a significant recurrence risk persisted even if MRD-negative after neoadjuvant treatment clearance. Event-free survival was 62% at 4 years with ctDNA clearance compared to 42% without clearance [45]. In the AEGEAN trial, a tumor-informed MRD-negative assay after neoadjuvant chemo-immune treatment was associated with a 3-year disease-free survival of 90% if associated with a complete pathologic response but only 70% if not associated with a complete pathologic response, indicating complexity of recurrences beyond MRD results in NSCLC [54]. In the NADIM trial, 92.3% of patients who cleared their ctDNA assessed with a plasma-informed assay were alive at 5 years compared to 59.2% who did not clear ctDNA [55].

Post-surgical MRD results and clinical outcomes have been reported in neoadjuvant trials assessed with a tumor-informed assay or a plasma-informed methylation assay. In the AEGEAN trial, disease-free survival at an early follow-up of 12 months was 89.3% in tumor-informed assay landmark MRD-negative patients compared to 14.3% in landmark MRD-positive patients [56]. In the NADIM II trial, plasma-informed methylation assay landmark MRD-negative patients had an event-free survival of 75% at 3 years even with neoadjuvant chemo-immune treatment and surgery. A follow-up of these patients with at least two successive MRD-negative results, which would reflect longitudinal and not landmark MRD testing, expectedly did better with only 4% (1 out of 25) relapsing [57].

Landmark MRD-positive reflects micrometastatic NSCLC needing perioperative systemic RX whether neoadjuvant treatment or not. However, landmark MRD-negative patients still carry a risk of recurrence that can benefit from perioperative chemo-immune or specific targeted treatment. Landmark MRD-negative patients will have more favorable outcomes than MRD-positive patients irrespective of receiving neoadjuvant/adjuvant treatment but will achieve their highest curative outcomes with perioperative systemic treatment. Delaying ‘adjuvant’ therapy unless and/or until MRD-positive with longitudinal testing in these patients is no longer ‘adjuvant’ treatment; it is metastatic NSCLC treatment associated with metastatic treatment outcomes.

## 7. Tumor-Informed or Plasma-Informed MRD Assay Testing?

Few direct comparisons of tissue-informed and plasma-informed assays in the same patient have been published. In indirect comparison with pooled data in the meta-analysis of 30 MRD trials in resected NSCLC, landmark testing with tumor-informed MRD assays demonstrated higher NPV of 83% compared to tumor-agnostic assays NPV of 77%. Landmark PPV was also higher in tumor-informed assays, 79% compared to 65% in tumor-agnostic assays. In longitudinal testing, NPV with tumor-informed assays was 90% compared to tumor-agnostic assays at 78% and PPV tumor-informed assays at 84% with tumor-agnostic assays 73% [29].

In a direct comparison study in 86 patients with resected NSCLC, a personalized assay with deeper sequencing of up to 20 variants outperformed the 338-gene tumor-naïve assay. Landmark NPV was 81.7% with the personalized assay compared to 75% with the tumor-naïve assay. In the longitudinal setting, tumor-informed NPV was 96.7% compared to 89.1% with the tumor-naïve assay, with PPV of 100% and 90%, respectively [44].

Clinical trials with indirect and direct comparison of tumor-informed and plasma-informed MRD testing have demonstrated higher NPV and PPV with tumor-informed assays. However, testing has been limited to just assessing prognostic endpoints of recurrence risk with landmark testing with either MRD assay still carrying a 20–25% NPV recurrence risk for individuals with resected NSCLC. MRD studies with either MRD assay have yet to evaluate whether MRD detection can change and improve patient outcomes.

Clinical utility differences of tumor-informed and plasma-informed MRD assays are contrasted in Table 2.

The cost of MRD testing is not assay-type-dependent but outcomes-dependent. Costs of commercial vendor tumor-informed MRD assay and plasma-informed NGS assay are similar. A favorable cost–benefit scenario with an ultrasensitive MRD-negative assay could be the certainty of no recurrence obviating the need for extensive surveillance imaging beyond annual screening low-dose chest CT. Conversely, an unfavorable cost–benefit scenario could result from a prolonged lead time of MRD detection sparking more frequent imaging without treatment outcomes benefit. However, a scenario of tumor-informed MRD detection and plasma-informed NGS assay identifying the molecular tumor biology could have a very favorable cost–benefit provided MRD detection-based treatment does result in improved survival outcomes. Until and unless that is true, arbitrary MRD testing with surveillance imaging carries an unfavorable cost–benefit analysis.

## 8. Do Tumor-Informed MRD Assays Assess Precision Molecular Tumor Biology?

Tumor-informed MRD assays have a different clinical utility than plasma-informed assays. Tumor-informed MRD assays are not designed to identify or denote specific somatic genomic alterations to guide precision oncology in the recurrence setting. They are limited to the initial primary tumor clones and just report the binary presence or absence of ctDNA as mean tumor molecules per ml (MTM/mL), parts per million (PPM), or reporting just ‘detected’ or ‘not detected’. They do not assess the clonal evolution of recurrence/metastatic clones and do not provide molecular tumor biology specifics. Tumor-informed MRD assays are solely designed for sensitive detection of clonal persistence of a resected cancer.

The larger plasma-informed NGS assays are designed to assess all of the specific ctDNA/RNA alterations present, whether from the primary tumor or metastatic clones. Plasma-informed NGS assays can identify cancer persistence and as well as the clonal evolution tumor biology of cancer recurrence to guide precision oncology treatment.

## 9. Is There an MRD VAF Threshold of Recurrence?

In the overt radiographic cancer setting, higher plasma ctDNA VAF carries a poorer prognosis across all stages of NSCLC with metastatic NSCLC typically having higher ctDNA VAF than earlier stages [32]. Plasma ctDNA shedding in resectable-stage NSCLC is also associated with poorer outcomes even with perioperative systemic treatment. In the phase II NADIM trial of neoadjuvant chemo-immune therapy in stage III NSCLC, a VAF threshold of 1% was associated with significantly poorer outcomes with the same treatment [58].

Tumor-informed assay VAF LODs range from 0.01% (100 PPM ctDNA) to as low as 0.001% (10 PPM ctDNA). Plasma-informed assays have higher LOD in the 0.1% (1000 PPM ctDNA) range down to 0.01% (100 PPM ctDNA). Developed ultrasensitive MRD assays report LOD down to 0.0001–0.0003% (1–3 PPM ctDNA). The current tumor-informed MRD assays are associated with lead times of months before overt radiographic recurrence. VAF are not usually subsequently reported comparing occult MRD recurrence versus radiographic recurrence.

Lower VAF LOD is associated with longer lead times. Zhang et al. reported MRD VAF > 0.55% was associated with a universal radiographic recurrence within 6 months compared to a VAF ≤ 0.55% with a median radiographic recurrence of 12 months [36].

A retrospective study testing banked plasma samples in a variety of solid tumor patients supports the lower the VAF, the longer the lead time before an overt cancer may be identifiable and that VAF will increase at the time of overt cancer detection. Plasma ctDNA VAF detection ranged from undetectable to 0.05–0.33% 3-plus years before clinical diagnosis, with higher VAF of 0.33–26.26% at the time of confirmed diagnosis [59]. Stage was reported as unknown in most of the patients.

The ultrasensitive very low levels of MRD detection have brought new clinical issues in MRD detection. Patients who are MRD-negative by ultrasensitive LOD assays appear to have survival outcomes compatible with cured cancer. However, patients with ultrasensitive MRD assay positive results can still have significantly prolonged survival outcomes. An ultrasensitive tumor-informed MRD detection assay with an LOD of 0.0001–0.0003% (1–3 PPM ctDNA) assessed in resected early-stage NSCLC performed better than the sensitive tumor-informed TRACERx assay with an LOD of 0.008% (80 PPM ctDNA) evaluating the same data set. The ultrasensitive assay identified ctDNA in 57% of stage I lung adenocarcinomas compared to 13–14% with the TRACERx assay and 79% stage II adenocarcinomas compared to 44%. Patients with pre-operative negative ctDNA at the ultrasensitive assay had 5-year overall survivals of 100% compared to 61.4% with ctDNA below the median and 48.8% with ctDNA above the median. Patients with any ctDNA detected <VAF 0.008% (80 PPM ctDNA) still had a 5-year survival of greater than 50% [60]. In post-operative landmark MRD samples with the same ultrasensitive assay, 18% were noted to be positive at below 10 PPM ctDNA. Those patients had a higher risk of recurrence and death than patients with undetectable ctDNA. However, there was a threshold of ultrasensitive LOD and outcomes. Patients with <10 PPM ctDNA had significantly improved relapse free survival (HR 0.38; *p* = 0.00086) and overall survival (HR 0.37; *p* = 0.021) compared to patients with ≥10 PPM ctDNA [61].

A pre-surgery personalized CAPP-Seq assay demonstrated significantly different outcomes in stage I NSCLC with a median VAF of 0.0031% (31 PPM ctDNA) threshold cutoff. The study found that 50% of stage I, 38% of stage II, and 7% of stage III patients had ctDNA levels of less than 0.01%. Patients above that median had a poorer relapse free survival (HR 3.69; 95% CI: 1.04–13.19) and poorer freedom from metastasis (HR 7.27; 95% CI: 1.99–26.55) than those below the median ctDNA VAF. Notably 90% of lung adenocarcinoma patients below that median of 31 PPM ctDNA remained metastases free at 5 years [62].

Higher VAFs are associated with more aggressive cancers and earlier post-surgical recurrences. Lower VAF is associated with a longer lead time of radiographic recurrence. VAF will increase over time and is typically higher upon radiographic recurrence. Patients with ultrasensitive MRD detection appear to still have a threshold of recurrence risks with more than half of these patients remaining metastases free with 5-year survival outcomes. This leaves MRD assay LOD differences moot unless treating longitudinal MRD-positive patients before overt radiographic recurrences improves survival outcomes.

## 10. Does MRD Detection Lead Time Matter?

Beyond technology leading to the development of new MRD assays with ultra-sensitive levels of ctDNA detection, the use of longitudinal MRD testing in the clinic still has unclear benefits for individual patients. Surveillance MRD-positive results heralds a recurrence but, if a thorough imaging assessment is negative and there are no symptoms, does treatment of an asymptomatic radiographic negative MRD recurrence improve survival outcomes? If it only sparks closer interval monitoring the potential impact may be lost as standard guideline recommended surveillance imaging to identify early asymptomatic recurrences have not been shown to impact group survival outcomes in NSCLC [63]. The survival impact answer of treating MRD recurrences is unknown but is the most pressing question of true patient benefit of MRD monitoring.

The closest clinical data to begin to answer the question of whether earlier treatment of recurrent NSCLC comes from a web-based patient reported symptoms trial that fast tracked patients upon reporting symptoms to immediate follow-up imaging compared to the routine scheduled follow-up visit and scheduled imaging. Those patients fast tracked as soon as they reported symptoms had a better performance status at the time of starting treatment, had a mean 5-week earlier start of systemic treatment and had an 8-month median and doubled 2-year overall survival benefit [64]. However, this far from answers the MRD-detected treatment question as this study reflected earlier treatment of overt cancer recurrence.

## 11. Conclusions and Future Directions

MRD testing technology continues to advance. However, the benefit of MRD assays will continue to have questions of limitations in the clinic for individual patients with resected NSCLC. It is important to recognize the impact of tumor biology and clonal evolution limitations on tumor-informed assay MRD testing and the lower sensitivity and potential false-positive findings with plasma-informed assays. The clear distinction of the different clinical utility and patient outcomes impact between landmark and longitudinal MRD testing is vital to understand what MRD results may or may not mean for individual patients.

Tumor-informed assay landmark MRD-negative does not provide certainty of a cured resected NSCLC and does not preclude an adjuvant treatment benefit. Studies with landmark MRD-negative tumor-informed assays in resected NSCLC consistently demonstrate a potential recurrence risk of up to 20% over time. Studies assessing immune-based and targeted therapies have shown a survival outcome benefit with adjuvant treatment even if a tumor-informed landmark MRD assay is negative, with the treated MRD-negative patient’s survival outcomes exceeding the MRD-positive treated patients. Clinical trials have yet to identify any escalation or de-escalation strategies of perioperative systemic therapy in NSCLC. The proven benefit of adjuvant treatment in resected NSCLC is also post-surgical window time dependent. Avoiding adjuvant treatment in the curable post-surgical window in landmark MRD-negative patients will miss potential curative outcomes for individuals who ultimately recur. Delaying perioperative systemic therapy until MRD-positive becomes metastatic treatment, not adjuvant treatment, with metastatic NSCLC outcomes and not adjuvant treatment outcomes.

Longitudinal MRD-negative does provide a deeper level and potentially earlier confirmation of a curative outcome than surveillance imaging. However, that curative outcome is still dependent upon time. Negative imaging over the same time will identify similar curative outcomes. Landmark MRD-negative can become longitudinal MRD-positive over time. The Zhang et al. study with 43-month follow-up identified a peak of longitudinal MRD-positive conversion at 18 months post-surgery, with only 2 of 55 patients converting from MRD-negative to positive after 2 years [36]. If undertaken, longitudinal surveillance MRD monitoring needs to be ongoing as long as the risk of MRD conversion persists. MRD testing does not preclude the need for imaging as tumor-informed MRD-negative CNS, intra-thoracic, second lung primaries, and potentially other low-tumor volume non-shedding oligometastatic recurrences amenable to local ablative treatment may still occur.

Advances in MRD technology are achieving deeper VAF LOD detection. This may paradoxically result in confounding clinical issues. Ultrasensitive MRD assays provide better sensitivity of landmark MRD-negative patient outcomes. However, ultrasensitive LOD MRD detection levels are associated with longer radiographic recurrence free intervals. In pre-surgery studies utilizing ultrasensitive MRD assays, half of patients with resected NSCLC did not have a recurrence within 5-years of follow-up. This may reflect a re-balancing of a too short follow-up interval with a longer lead time of recurrence but also brings into question whether ultra-sensitive LOD could be reflecting a different tumor biology and recurrence potential based upon a certain threshold LOD. Ultrasensitive MRD assays may present a potential paradox of a stronger NPV but a weaker PPV.

Another clinical issue is the potential lack of or delayed precision oncology treatment approach with any tumor-informed MRD assay. Beyond the tumor-informed assay clonal limitations and the overarching question of whether any treatment at the time of an MRD detected recurrence changes a patient’s survival outcome, ultrasensitive LOD detection could further confound precision oncology treatment. Ultrasensitive MRD LOD detection would occur much earlier than ctDNA detection with a broader but higher LOD plasma-informed NGS assay. This leaves the same clinical enigma of tumor-informed assays lacking and ultrasensitive LOD further delaying identification of the needed molecular tumor biology to guide precision oncology. It is clear in the overt metastatic setting that initiating systemic treatment without knowing the molecular tumor biology is fraught with significantly poorer outcomes [65]. Initiating systemic treatment without plasma-informed assay identification of the molecular tumor biology would also be treating without knowing the tumor biology.

An MRD detection signal prompting closer clinical awareness and imaging follow-up could be a path to earlier identification and treatment of recurrent NSCLC. However, that is a far different impact than the fundamental clinical questions of MRD testing that remain unanswered. Do MRD-positive recurrences have a different tumor biology than overt radiographic recurrences? Does systemic treatment at the time of an MRD detection recurrence with immune-based therapy or targeted therapies improve patient survival outcomes?

Clinical trials to validate the outcomes benefit of MRD testing for patients will need to be designed beyond the traditional imaging-based response rate and progression free survival outcomes and encompass MRD ctDNA response and MRD-negative control durability endpoints. Randomized treatment arms will need to be at several levels based upon tumor-informed MRD assay detection, plasma-informed NGS negative/positive and imaging findings. Considered randomized treatment arms should include patient subsets: (i) MRD detected with imaging negative and plasma-informed NGS assay negative treated with immune-based systemic treatment; (ii) MRD detected with imaging negative with plasma-informed NGS assay positive guided precision oncology treatment; and (iii) MRD detected with plasma-informed NGS assay negative or positive with standard of care treatment only upon a radiographic recurrence.

Cautionary tumor biology and clinical issues remain in the clinical utility of MRD testing in resected NSCLC. These need to be clarified with certainty before MRD testing can be an absolute clinical guide to meaningfully impact individual patient outcomes. Advancing technology of MRD assays per se does not improve individual patient outcomes unless that MRD testing can improve individual patient outcomes. MRD testing studies must go beyond focusing on specific MRD assay technology and the redundant prognostic validation of plasma ctDNA and demonstrate individual patient outcome benefits based upon MRD detection before MRD guided NSCLC management should step beyond a technology driven concept to a patient outcomes driven approach.

## Figures and Tables

**Table 2:** Clinical differences between tumor-informed and plasma-informed MRD assays. (LOD = level of detection.).

Tumor-Informed Assay	Plasma-Informed Assay
Smaller gene panels limited to primary tumor alterations	Larger gene panels
Ultrasensitive LOD	Less sensitive LOD
May be limited by tissue spatial heterogeneity	May be limited by potential false positive cell-free DNA findings
Will not assess parallel metastatic clones or cancer recurrence clonal evolution	Can assess parallel metastatic clones and cancer recurrence clonal evolution
Optimized for MRD detection; does not identify molecular tumor biology	Optimized to identify molecular tumor biology
Can not guide precision oncology treatment approaches	Can guide precision oncology treatment approaches

## Data Availability

No new data were created or analyzed in this study. Data sharing is not applicable to this article.
